# Active surveillance identified a neglected burden of macular cases of Post Kala-azar Dermal Leishmaniasis in West Bengal

**DOI:** 10.1371/journal.pntd.0007249

**Published:** 2019-03-11

**Authors:** Ritika Sengupta, Surya Jyati Chaudhuri, Srija Moulik, Manab Kumar Ghosh, Bibhuti Saha, Nilay Kanti Das, Mitali Chatterjee

**Affiliations:** 1 Department of Pharmacology, Institute of Post Graduate Medical Education and Research, Kolkata, West Bengal, India; 2 Govt. Medical College, Purulia, West Bengal, India; 3 Department of Tropical Medicine, School of Tropical Medicine, Kolkata, West Bengal, India; 4 Department of Dermatology, Bankura Sammilani Medical College, Bankura, West Bengal, India; Universidade do Estado do Rio de Janeiro, BRAZIL

## Abstract

**Background:**

Post Kala-azar Dermal Leishmaniasis (PKDL) develops in patients apparently cured of Visceral Leishmaniasis (VL), and is the strongest contender for being the disease reservoir. Therefore, existence of a few cases is sufficient to trigger an epidemic of VL in a given community, emphasizing the need for its active detection and in turn ensuring success of the current elimination program. This study explored the impact of active surveillance on the demographic profile of PKDL patients in West Bengal.

**Methodology/Principal findings:**

Patients with PKDL were recruited through passive (2003-date, n = 100) and active surveillance (2015-date, n = 202), the former from outpatient departments of dermatology in medical colleges in West Bengal and the latter through an active door-to-door survey in four VL hyper-endemic districts of West Bengal. Passive surveillance indicated a male preponderance and a predominance of polymorphic lesions, whereas active surveillance indicated absence of any gender bias and more importantly, macular PKDL constituted almost 50% of the population burden. In terms of polymorphic vs. macular PKDL, the former appeared at a later age, their disease duration was longer and had a higher parasite burden. In the polymorphic variant, the lesional distribution was asymmetrical, comprised of papules/nodules/macules that were present mainly in sun-exposed areas whereas in macular cases, the hypopigmented patches were diffusely present all over the body.

**Conclusions/Significance:**

Active surveillance unraveled a disease component whose demographic profile showed important differences with PKDL cases who sought treatment in government hospitals. Detection of a higher proportion of macular cases indicates that this variant is not an uncommon presentation as conventionally stated in text books, and should be studied in greater detail to ensure success of the ongoing Leishmaniasis elimination programme.

## Author summary

Post Kala-azar Dermal Leishmaniasis (PKDL) is a dermal condition that occurs in South Asia in 10–20% of patients after apparent cure from Visceral Leishmaniasis (VL). It presents with macular or papulonodular lesions that harbor *Leishmania* parasites, thus playing a major role in the transmission cycle. Accordingly, development of strategies for case finding, diagnosis, and treatment are major foci of the Leishmaniasis elimination programme. The major limitations include a risk of misdiagnosis, costly case finding along with inadequate and/or unnecessary treatment. This study delineated changes in the epidemiological characteristics of PKDL cases in West Bengal arising out of two modes of surveillance. Traditionally, passive surveillance was the major mode of patient recruitment, but from 2015, active surveillance came into effect. This led to the scenario changing drastically, with important differences appearing in the demographic profile of PKDL. Active surveillance proved to be a game changer as it unravelled a huge disease burden of macular cases, which had so far remained undetected. Therefore, contrary to previous studies, our study has established that macular PKDL in West Bengal, and perhaps India, is not a rare, uncommon presentation, and therefore warrants a deeper analysis.

## Introduction

Leishmaniasis are a group of neglected tropical diseases caused by the parasite *Leishmania* and demonstrates clinical pleomorphism with regard to the causative species, disease reservoirs, vectors as also host-immune responses. It can manifest as life-threatening and/or disfiguring lesions ranging from innocuous self-healing cutaneous lesions to fatal visceralization or a dermal dissemination [1]. Post Kala-azar Dermal Leishmaniasis (PKDL) usually presents in patients with a history of treated Visceral Leishmaniasis (VL)/ kala-azar caused by *L*. *donovani*. It is unique to South Asia and East Africa (mainly Sudan) with approximately 5–10% of apparently cured VL patients developing PKDL in South Asia as against 50–60% in East Africa [2, 3]. Unlike VL where patients have significant morbidity and present with prolonged fever, hepatosplenomegaly, weight loss and anemia, patients with PKDL manifest with innocuous hypopigmented macular lesions (macular PKDL) or a combination of macules/papules/nodules (polymorphic PKDL). It is possibly the most challenging variant of Leishmaniasis, especially in terms of its etiopathogenesis [4–6]. The clinical diagnosis of PKDL remains empirical and till now, was mostly dependent on self-reporting i.e. passive surveillance [7]. As PKDL is a disease of the poor, occurring mostly in remote rural villages with poor housing and little or no access to modern health-care facilities and importantly, is not life-threatening, the tendency to actively seek treatment is minimal [8].

In South Asia, the VL endemic zones include the Gangetic plains of India, Bangladesh, and Nepal. In 2005, the governments of Bangladesh, India, and Nepal agreed on a regional initiative, the Regional Kala-azar Elimination Programme (KAEP) to eliminate VL as a public health problem from the region by 2010. This was further extended to 2015, the target being to reduce the annual incidence of kala-azar to less than one new case in a population of 10,000 at a district (or sub-district) level [9]. This target was later extended to 2017, which too has been missed, and presently stands at 2020 [10].

PKDL cases harbor parasites within their dermal lesions, and as they are easily accessed by sandflies, play a major role in the transmission cycle, especially as VL is anthroponotic, emphasizing their inclusion as a component of the ongoing VL elimination programme [11]. PKDL is recognised as a constraint in the Leishmaniasis elimination effort, and accordingly, development of strategies for case finding, diagnosis, and treatment are among the major objectives of the KAEP [11]. However, the strategies are currently not based on solid scientific evidence, limitations being costly case finding, risk of misdiagnosis, and possibility of inadequate and unnecessary treatment with potentially toxic drugs [11]. From 2015 onwards, an ‘active case surveillance’ approach was adopted in the VL/kala-azar hyper endemic districts of West Bengal. This translated into a dramatic rise in the number of PKDL cases [12] and significant changes in the demographic profile of the disease warranting a deeper analysis of the various clinical and epidemiological aspects of PKDL. This study aimed to delineate the differences, if any, in the demographics of PKDL cases detected by active vs. passive surveillance over the last 15 years in West Bengal.

## Materials and methods

### Study population

From 2003 to date, patients clinically diagnosed with PKDL were recruited through passive surveillance from the Dermatology outpatient departments of the School of Tropical Medicine/Calcutta Medical College/ Institute of Postgraduate Medical Education and Research, Kolkata, West Bengal. Additionally, from 2015 onwards, active field surveys were conducted in VL endemic districts of West Bengal (Malda, Dinajpur, Darjeeling and Birbhum) by a camp approach, wherein an initial house-to-house survey was conducted by first-line health workers [Kala-azar Technical Supervisors and ASHA workers (accredited social health activists)] using standard case definitions and defined risk factors, for example, living in an endemic area and having an epidemiological link (past history of VL). The suspected cases were then examined at medical camps held at block hospitals. These camps were conducted by a team of 4–10 members comprising of medical officers, laboratory technicians, health supervisors, health workers and community based health volunteers. The suspected cases were assessed clinically, rk39 strip test and if positive, informed consent was taken for a 4 mm punch biopsy.

In general, cases with hypopigmented macules were considered as macular PKDL, whereas cases with an assortment of papules, nodules, macules, and/or plaques were termed as polymorphic PKDL. The cases were included only when confirmed by internal transcribed spacer-1 (ITS-1) PCR [13] and/or Giemsa staining of dermal biopsies. None suffered from any co-infection or pre-existing disease. Owing to the teratogenic potential of miltefosine, and PKDL being a non fatal disease, pregnant women were not offered any treatment and treated after completion of gestation and lactation period. After confirmation by ITS-1 PCR, their parasite load was quantified by amplification of kinetoplastid DNA [12]. Cases were randomly allocated, as per the existing guidelines, to receive Miltefosine (for >25 kg b.w.100 mg, p.o. x 12 weeks and 50 mg p.o. x 12 weeks for <25 kg) or Liposomal Amphotericin B, LAmB (5 mg/kg body IV, twice weekly, for 3 weeks) [14].

### Measurement of parasite load by real-time polymerase chain reaction

A defined number of *Leishmania* parasites sourced from an *L*. *donovani* strain (ranging from 10 to 1 × 10^5^) was added to blood (180 μL) from a healthy control. Following extraction of DNA using a QIAmp DNA Mini kit (Qiagen, Hilden, Germany), real time -PCR was performed in an Applied Biosystem Step One Plus (Applied Biosystems, Foster City, CA, USA). A fragment of 116 bp of *L*. *donovani* kDNA was amplified by using a primer set (forward 5′-CCTATTTTACACCAACCCCCAGT-3′ and reverse 5′-GGGTAGGGGCGTTCTGCGAAA-3′). Template DNA (5 μL) was added to 19 μL reaction mixture containing SYBR Green qPCR Master mix (Roche, Basel, Switzerland) and 400 nM of each primer. For measurement of parasite load, a standard curve was generated as previously described [12]. Negative controls included DNA from a healthy donor (no amplification), and a reaction mixture with water instead of template DNA (no-template control [NTC]). The range of the kDNA qPCR assay had an efficiency of 5 orders of magnitude (1x10^5^ to 1x10 parasite/μg of genomic DNA). The standard curve (ranging from 10 to 1 × 10^5^) had a mean square error of 0.007, correlation coefficient, r^2^ = 0.995. The number of parasites was extrapolated from the standard curve and parasite load stated as the number per μg genomic DNA. As the parasite number when <10 reported a cycle threshold (Ct) value almost equivalent to NTC, it was accorded an arbitrary value of 1.

### Statistical analysis

Results were expressed as median (Interquartile range, IQR) and data analyzed between groups by Kruskal wallis test followed by Dunn’s multiple comparison test for non-parametric data; where 2 groups were present, the Mann-Whitney test was used using GraphPad Prism software (version 5.0, GraphPad software Inc., La Jolla, CA, USA), p< 0.05 was considered significant.

### Ethics statement

The study received approval from the Institutional Ethics Committee of School of Tropical Medicine, Kolkata, India and Institute of Post Graduate Medical Education and Research, Kolkata, India. All experiments were performed in accordance with relevant guidelines and regulations. Individuals or their legally acceptable representative (if age was <18 years) gave a written informed consent. The patients in this manuscript have given written informed consent (as outlined in the PLOS consent form) to publication of their case details.

## Results

### Study population

**Fig 1:**
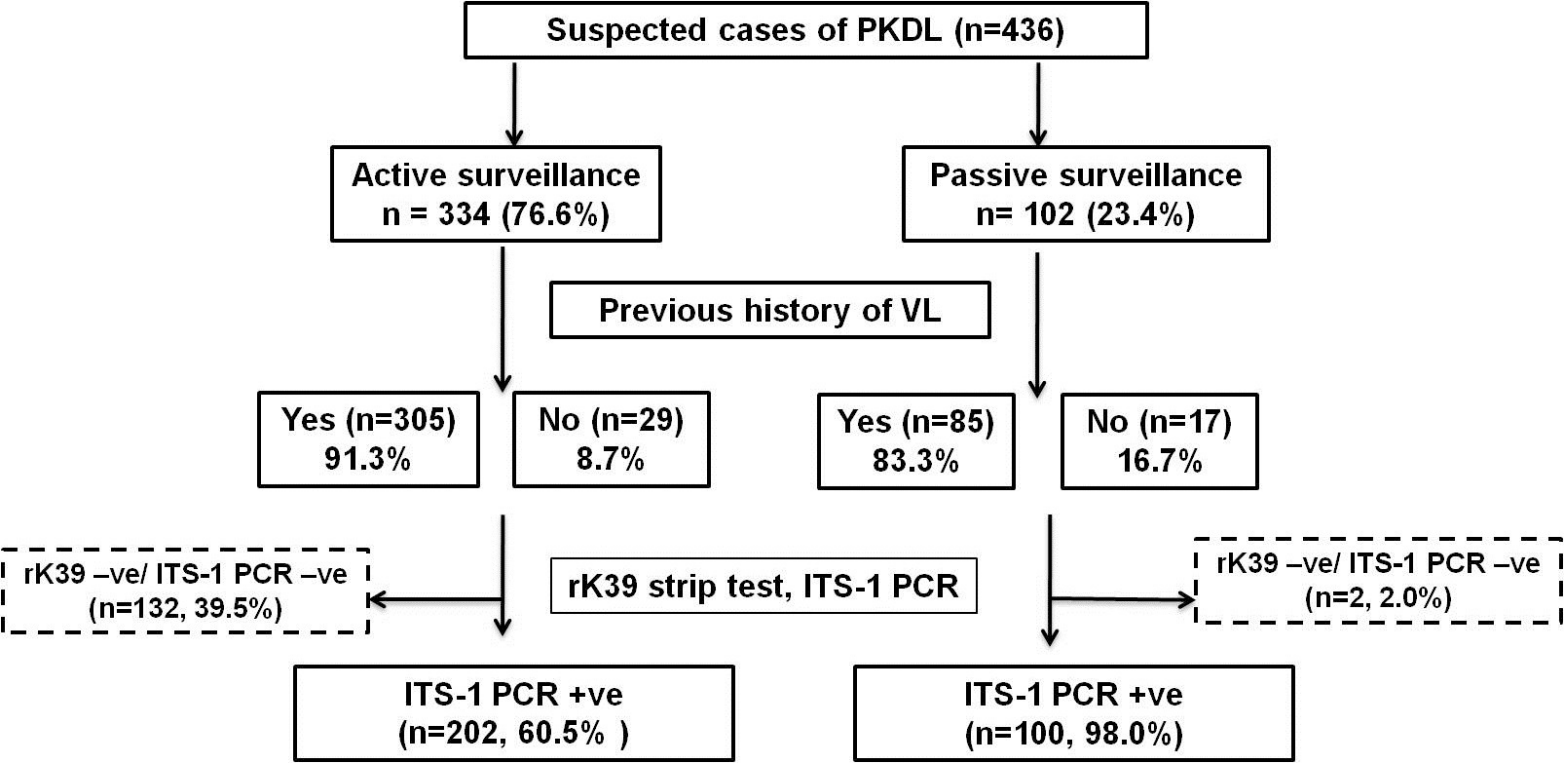
Schematic diagram indicating total number of patients recruited following active or passive surveillance in West Bengal. PKDL: Post Kala-azar Dermal Leishmaniasis, VL: Visceral Leishmaniasis, -ve: negative, +ve: positive.

Since 2003, irrespective of the surveillance, 436 suspected cases of PKDL were registered, and included 102 via passive and 334 following active surveillance (**Fig 1**). Following passive surveillance, 83.3% gave a history of VL while 100/102 (98%) were confirmed as cases of PKDL (**Fig 1**); amongst these 100 confirmed cases, 15 (15%) reported no history of VL. Following active surveillance undertaken in the VL/kala-azar hyper endemic districts of Malda, Dinajpur, Darjeeling and Birbhum from 2015–2018, a latent disease burden of 334 suspected cases of PKDL were identified, with 91.3% having a previous history of VL. Following their initial screening by KTS workers, they were physically examined at a ‘medical camp’ and 202 (60.5%) were confirmed as PKDL by ITS-1 PCR (**Fig 1**). Of these confirmed cases, 16 (7.9%) did not report any prior history of VL. There were 5 rK39 -ve cases (2 from passive surveillance and 3 from active surveillance), but were ITS-1 PCR +ve. The rK39 -ve/ ITS-1 -ve cases (n = 132), were followed up, and their skin lesions identified as pityriasis versicolor, vitiligo or pityriasis alba.

### PKDL cases predominantly resided in VL-endemic areas

**Fig 2:**
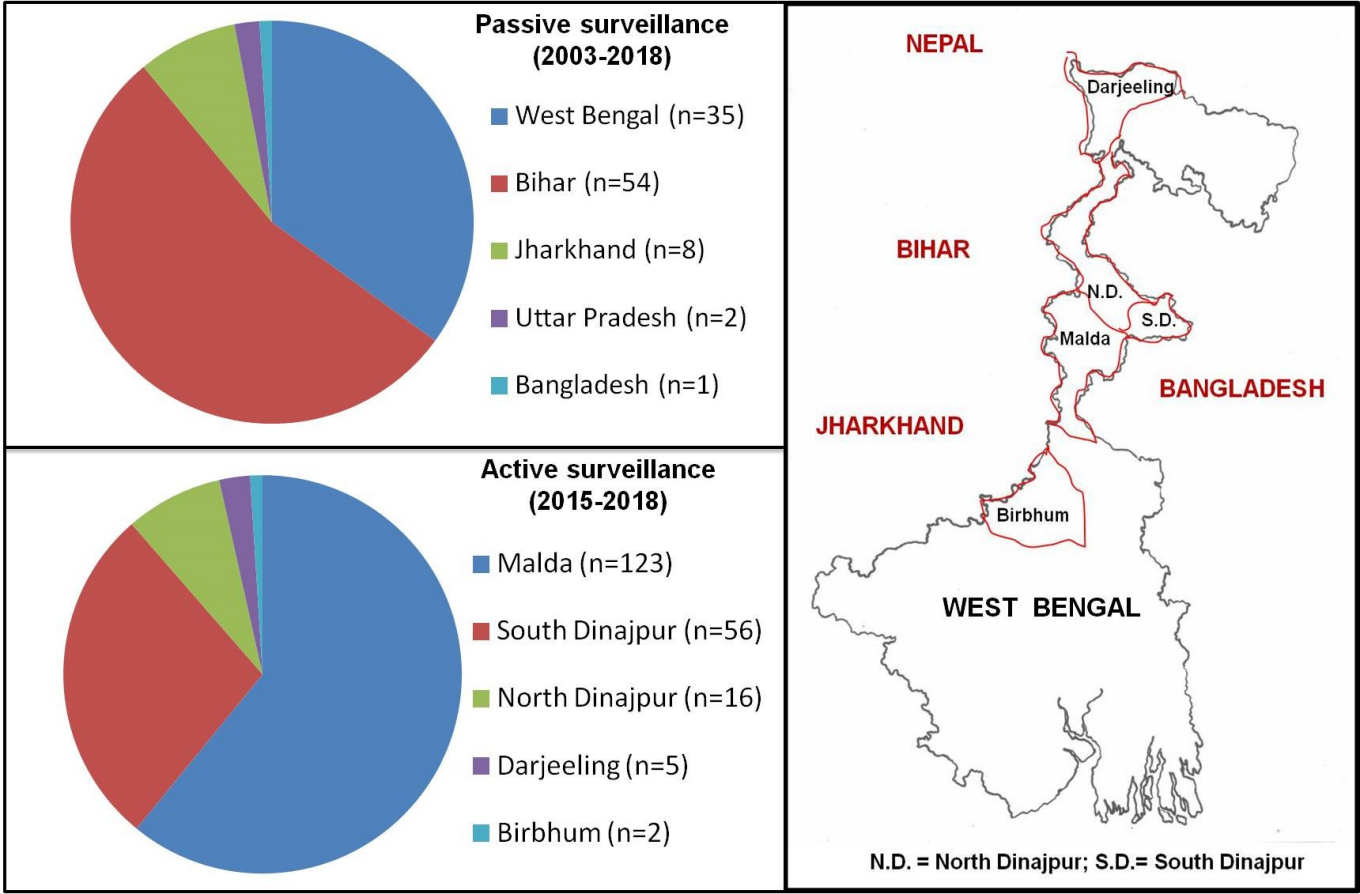
Geographical pie distribution of PKDL cases recruited through passive and active surveillance. A map of West Bengal highlighting the VL-endemic districts from where patients were recruited during active surveillance.

During passive surveillance, cases (n = 100) were not only from West Bengal, but included the adjoining states of Bihar, Jharkhand and Uttar Pradesh as also Bangladesh; majority originated from Bihar (n = 54, 54%), while the rest were from West Bengal (n = 35, 35%), Jharkhand (n = 8, 8%), Uttar Pradesh (n = 2, 2%) and Bangladesh (n = 1, 1%), **Fig 2**. Of the 54 PKDL cases from Bihar, more than 50% (n = 30, 55.6%) were from districts with high VL endemicity and antimonial resistance [15,16] e.g. Samastipur, Muzzafarpur, Vaishali, Araria, Saran, Purnia and Saharsa, while the rest were from areas of low endemicity namely Siwan, Madhubani, Begusarai, Darbhanga, Kishenganj, Gaya, Jehnanabad, Khagaria and Aurangabad [17]. In West Bengal, patients reported not only from VL-endemic districts (Malda, Murshidabad, Birbhum, Dinajpur, Darjeeling, North 24 Parganas, South 24 Parganas, Nadia and Burdwan), but also from non-VL endemic districts (Kolkata, Howrah, Midnapore and Alipurduar).

Following 2015, active surveillance was initiated in four VL-endemic districts of Bengal namely Malda, North and South Dinajpur, Darjeeling and Birbhum that unraveled a huge disease burden (n = 202). These areas reported the maximum number of PKDL cases, as they share their western borders with the VL-endemic states of Bihar and Jharkhand, and eastern borders with Bangladesh. Malda was the leading district with the maximum number of cases (n = 123, 60.9%), followed by South Dinajpur (n = 56, 27.7%), North Dinajpur (n = 16, 7.9%), Darjeeling (n = 5, 2.5%) and Birbhum (n = 2, 1%), **Fig 2**. From January 2015- till date, 16 active field surveys were conducted in these VL endemic districts, namely Malda (6 visits) where 4 blocks were included [Habibpur, Gazole, Old Malda and Bamongola]. In South Dinajpur, 7 field trips were conducted in Kushmandi, Banshihari, Harirampur and Tapan blocks. One field trip each was undertaken in North Dinajpur (2 blocks, Karandighi and Goalkhopar-II), Darjeeling (2 blocks, Khoribari and Phasidewa) and Birbhum (1 block, Bolpur).

### Demographics of PKDL cases recruited through passive vs. active surveillance

**Fig 3:**
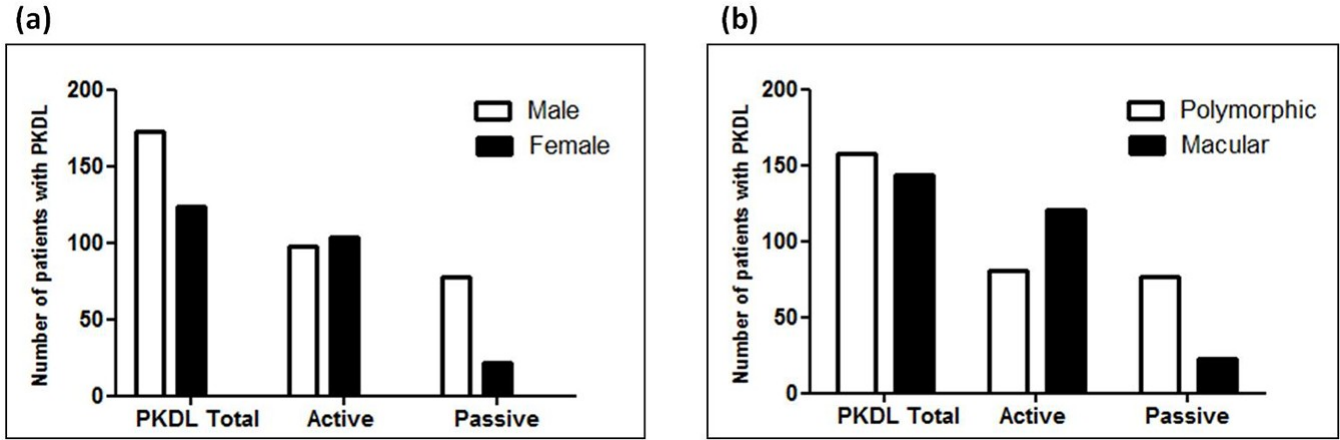
Impact of surveillance upon gender and lesional distribution in PKDL. **(a)** Bar diagram showing the overall distribution of males and females in the PKDL population, as also following passive or active surveillance **(b)** Bar diagram showing the overall distribution of polymorphic and macular lesions in the PKDL population, following detection by passive or active surveillance.

A male preponderance in PKDL reported during passive surveillance was in concordance with previous reports [4, 7, 18, 19] (**Table 1, Fig 3A**), whereas with active surveillance the scenario changed drastically as the ratio became 1:1.1, indicating absence of any gender bias (**Table 1, Fig 3A**). Following pooling of the data, the study demonstrated a male: female ratio of 1.3:1, with 57.3% (n = 173) being males and 41.1% (n = 129) females (**Table 1, Fig 3A**). Irrespective of the survey, the median age, disease duration and lag period, i.e. gap between manifestations of PKDL following completion of VL treatment were comparable (**Table 1**). Interestingly, the percentage of PKDL cases reporting a disease duration of less than 1 year increased following active surveillance as 40/202 (19.8%) had a disease duration of <1 year *vis-a-vis* 8/100 (8%) cases from passive surveillance. Out of 302 PKDL cases, parasite load was measured in 229, and on a stratification based on surveillance, cases from the passive survey had a 4.0 fold higher parasite burden than those recruited from active surveillance, p<0.01 (**Table 1**).

**Table 1:** Demographic profile of patients with PKDL.

	Overall N = 302	Passive surveillance N = 100	Active surveillance N = 202
Age (years)*	24(16.25–34)	25(18–35)	23(16–31.5)
**Sex (M:F)**	1.3:1	3.5:1	1:1.1
Disease duration (years)*	2(1–4)	3(1–6)	2(0.9–3)
Lag period (interval between cure of VL and onset of PKDL, years)*	3.2(2–6)	4(2–6)	3(1–7)
**Lesion type (Polymorphic: Macular)**	157:145 (1.1:1)	77:23 (3.3:1)	80:122 (1:1.5)
**Parasite load (parasites/**μg of genomic DNA)*	5417(918–47738)	17828(2271–69159)^@^	4471(784.5–35535)

*Values given in median(IQR); M: Male; F:Female

^@^p<0.01 as compared to active surveillance

With regard to the type and distribution of lesions, striking differences emerged depending on the type of surveillance. Following passive surveillance, there was an overwhelming predominance of the polymorphic variant *vis-a-vis* the macular (**Table 1**) whereas active surveillance unravelled a huge population having macular lesions, that translated to the ratio of polymorphic: macular shift dramatically to 80:122. Taken together, the cumulative scenario mimicked the active surveillance data, and was possibly a more accurate representation of the lesional distribution of PKDL, at least in West Bengal (**Fig 3B**).

As the patients enrolled during passive surveillance were from VL-endemic states of India and Bangladesh, it was relevant to examine if differences observed were attributable to their geographical location. Accordingly, they were sub-stratified into (i) West Bengal, n = 35 (ii) Bihar, n = 54 and (iii) other regions (Jharkhand Uttar Pradesh and Bangladesh), n = 11. Irrespective of the geographic location, the demographic profiles in all three groups was comparable, and hence were analyzed as a single group (S1 **Table**).

**Fig 4:**
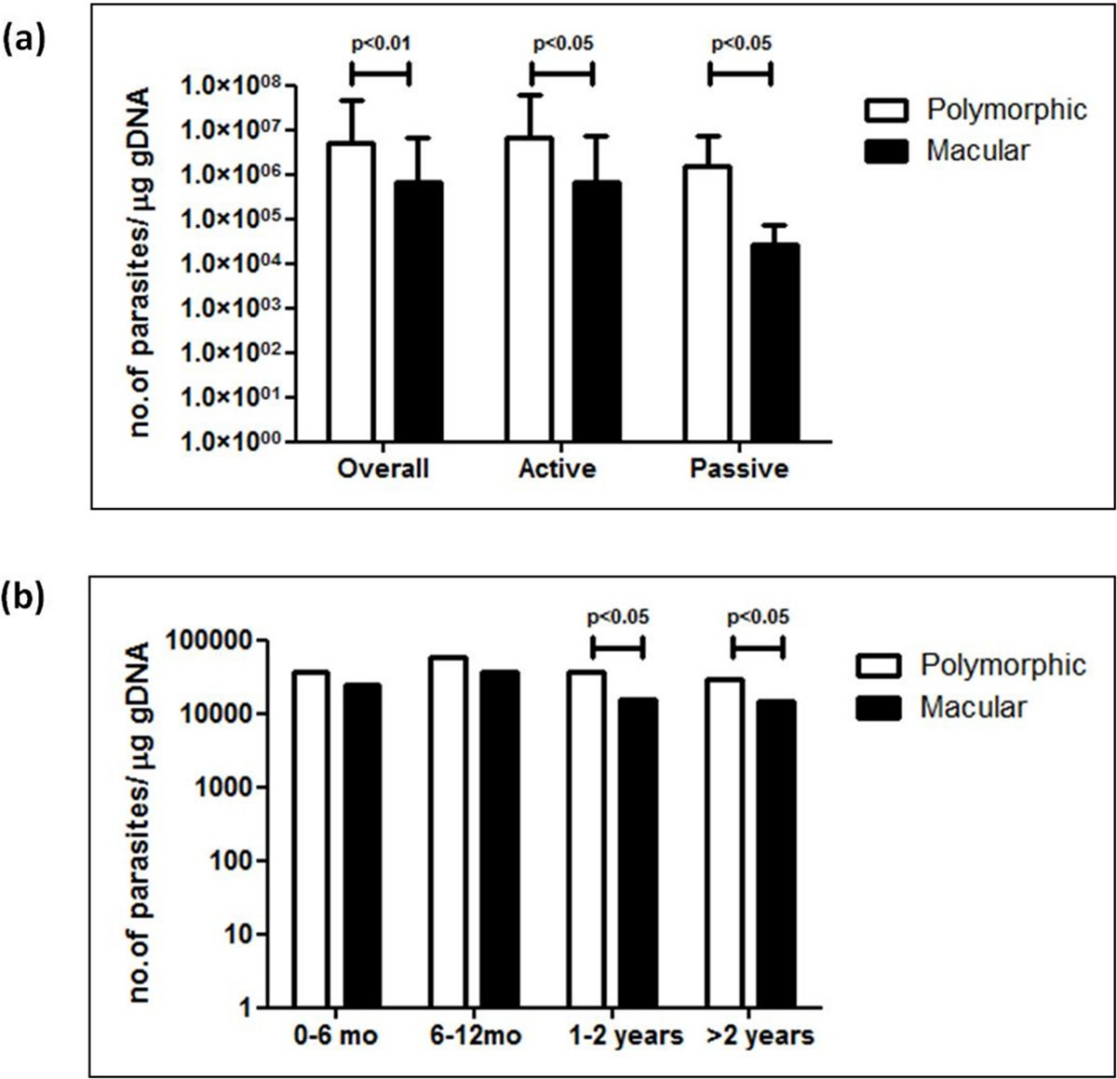
Effect of surveillance and disease duration on parasite load. **(a)** Bar diagram showing parasite burden in polymorphic and macular PKDL following passive and active surveillance [Values given in Mean ± SEM] **(b)** Bar diagram showing median parasite burden in polymorphic vs. macular PKDL with disease progression.

In terms of their lesional profile, significant differences emerged with regard to the median age, disease duration and lag period. Following passive surveillance, the median age (in years) for polymorphic PKDL was significantly higher than the macular group, and this difference persisted with active surveillance (**Table 2**). The gender bias present during passive surveillance was not evident following active monitoring. Additionally, upon stratification, in terms of their lesional profile, the significantly longer disease duration and lag period present in the polymorphic variant was absent following active case detection (**Table 2**). Furthermore, significant differences emerged regarding their parasite load as with passive surveillance, it was 3.0 fold higher in the polymorphic variant as compared to the macular variant, and this trend remained even with active surveillance, being 3.2 fold higher (**Table 2, Fig 4A**).

**Table 2:** Demographic profile of patients with polymorphic vs. macular PKDL recruited by passive or active surveillance.

	Passive surveillance		Active surveillance	
	Polymorphic PKDL	Macular PKDL	Polymorphic PKDL	Macular PKDL
Age (years)*	30(22–40)^@^	17(12–28)	30(22–40)^@@^	20(13–30.25)
**Sex (M:F)**	3.5:1	2.3:1	1.1:1	1:1.9
Disease duration (years)*	3(2–7)^#^	2(1–4)	2(1–3)	1.05(0.95–3)
Lag period (interval between cure of VL and onset of PKDL, years)*	3.25(2–9.75)^$^	1.5(1–4.5)	4(2–7)	4(2–6)
**Parasite load** **(parasites/**μg of genomic DNA)*	19426 (1532–89965)^#^	6554 (3204–16072)	27856 (651–90561)^##^	8537 (886–76893)

*Values given in median(IQR); M: Male; F:Female

^#^p<0.05

^$^p<0.01

^@^p<0.001 as compared to macular PKDL following passive surveillance

^##^p<0.05

^@@^p<0.001, as compared to macular PKDL following active surveillance.

### Variation in parasite load with disease progression in macular vs. polymorphic PKDL

Based on the disease duration at the time of reporting, they were categorised into 4 groups, namely 0–6 months, 6–12 months, 1–2 years and >2 years. For the initial 0–6 and 6–12 months, both variants showed a comparable median parasite load, being 36458 (730–642578) vs. 24365(958–504512) and 57785(450–154632) vs. 35825(489–65582) parasites/μg of genomic DNA respectively (**Fig 4B**). However, when the duration increased to 1–2 years, a 2.3 fold higher parasite load was obtained in the polymorphic vs. macular variant being 35854 (984–85524) vs. 15547 (458–45785), p<0.05 (**Fig 4B**). This trend persisted when the duration increased to >2 years, with the difference being 1.9 fold, 28544(657–55284) vs. 14652 (285–24658) parasites/ μg of genomic DNA), p<0.05 (**Fig 4B**).

### Distribution of lesions

**Fig 5:**
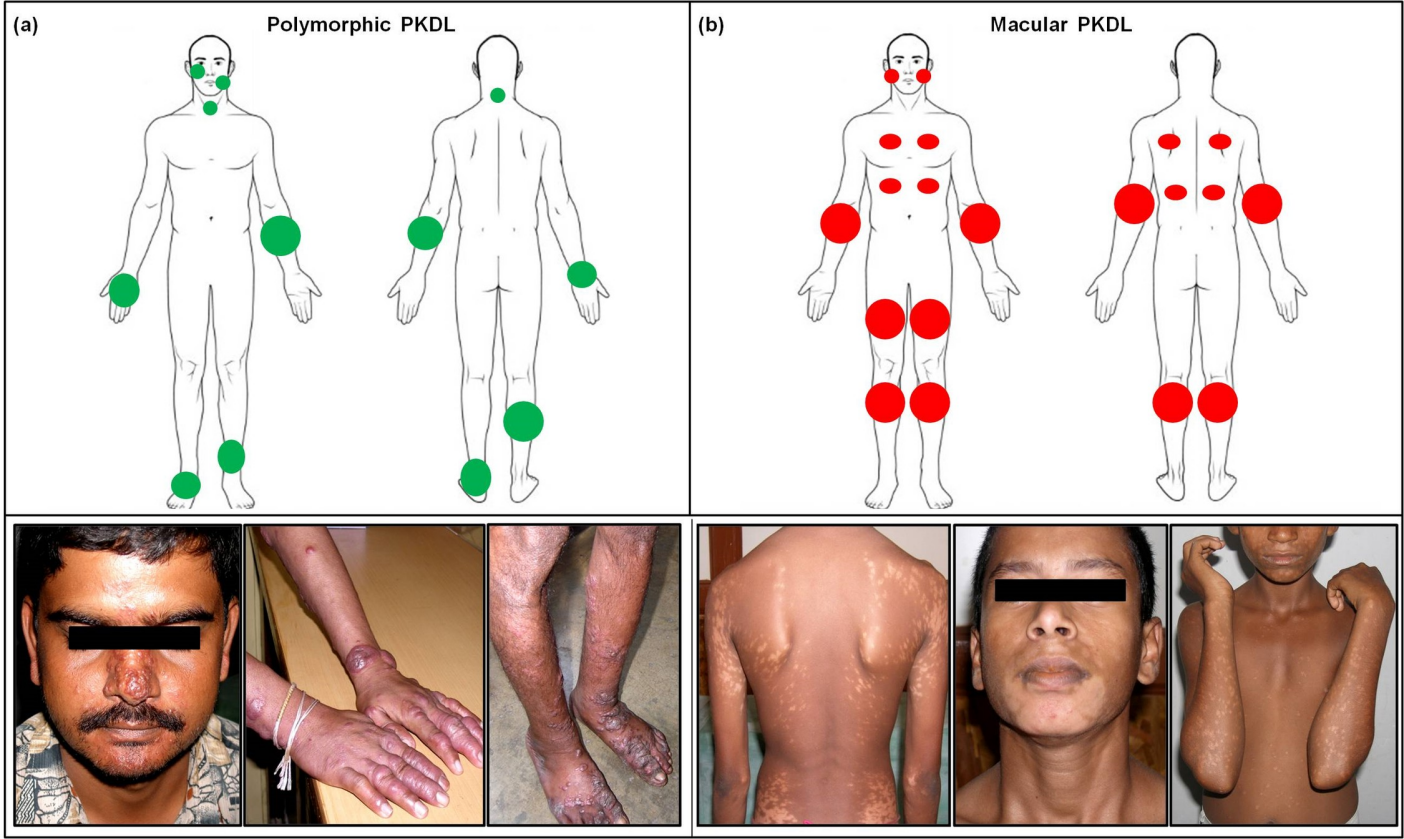
Distribution of lesions and clinical profile of patients with PKDL. **(a)** Diagrammatic representation showing the pattern of distribution of lesions in patients with polymorphic PKDL along with representative clinical profiles. **(b)** Diagrammatic representation showing the pattern of distribution of lesions in patients with macular PKDL along with representative clinical profiles.

Irrespective of the type of surveillance, differences existed in the lesional distribution pattern of polymorphic vs. macular PKDL. In polymorphic cases, lesions were asymmetrically distributed, varied from 8–12 in number and appeared mainly in sun exposed areas (**Fig 5A**). However, for macular PKDL, the distribution was diffuse, patchy, mostly symmetrical, with macules being sometimes large and coalescent (**Fig 5B**). In some patients, these patches developed initially in one area (e.g. upper limbs) as minute pin-point lesions and gradually spread to other areas (e.g. trunk). Based on the anatomical distribution, majority had lesions all over the body i.e. face, trunk and/or limbs followed by lesions on the face and neck, and least being on the face and trunk. Five patients reported mucosal involvement, where four had papules/nodules present on the lips, tongue, buccal mucosa and one on the glans penis [20].

### PKDL is a SAG driven phenomenon

As PKDL is proposed to be a SAG driven phenomenon [21], we examined the treatment profile during VL in 257/302 patients. Irrespective of the type of surveillance, the treatment modality for VL in the majority was SAG followed by Miltefosine, LAmB and herbal medicines (**Table 3**). The disease duration and lag period of cases who received SAG was comparable, being 4.5 (3–8) vs. 3.2(2–6.5) years and 4(3–7) vs. 3.7(2.2–6.0) years respectively.

**Table 3:** Treatment received by PKDL cases during VL.

Treatment received for VL (n = 257)	Passive Surveillance (n = 84)	Active Surveillance (n = 173)
SAG (Sodium Antimony Gluconate)	80 (95.2%) (P:M = 5:1)	130 (75.1%) (P:M = 1:1.3)
Miltefosine	3 (3.5%) (P:M = 2:1)	28 (16.1%) (P:M = 1:1)
LAmB (Liposomal Amphotericin B)	1 (1.2%) (P = 1)	14 (8.0%) (P:M = 1:1)
Others (herbal treatment)	-	1 (P = 1)

P = Polymorphic; M = Macular

## Discussion

Based on the limited resources invested in diagnosis, treatment, and control, and its strong association with poverty, Leishmaniasis is classified as one of the most neglected diseases. Disease burden estimates place leishmaniasis second in mortality and fourth in morbidity among all tropical diseases with over 1 billion people in endemic areas still at the risk of contracting one or the other forms of the disease [22, 23]. VL is a major public health importance in India, Bangladesh and Nepal and additionally, for reasons still unknown, a certain population of apparently cured VL patients go on to develop PKDL, whose clinical manifestations can be confused with other dermal disorders like leprosy, vitiligo, etc. The South Asian elimination initiative of VL and the 2012 London Declaration on neglected tropical diseases (NTDs) have raised global awareness about Leishmaniases and translated substantially into increased funding especially for control [1]. However, most of the classical challenges of a NTD persist and include limited therapeutic options, suboptimal diagnostics, and poor community awareness.

Historically the occurrence of VL in India has shown a cyclical pattern with case resurgence characteristically occurring every 15 years [24], but the cause remained poorly defined. Modelling data provided valuable information regarding the transmission of VL and suggested the potential causes for resurgence are asymptomatic individuals and in particular, PKDL cases [25]. Importantly, as PKDL is the only inter-epidemic reservoir of anthroponotic VL, with proven transmission potential [26], existence of even a few cases can trigger a new epidemic of VL, reiterating the need for the early diagnosis and prompt treatment of every case of PKDL [11].

The implementation of the ongoing elimination programme unravelled a different clinico-demographic profile of PKDL patients in West Bengal. Prior to 2015, passive surveillance was the sole mode of identification, with suspected cases having clinical symptoms similar to PKDL, a past history of VL, and they self-reported at the dermatology outpatient departments of different hospitals in Kolkata. These patients (n = 100, **Fig 1)** were not restricted to West Bengal, but included the VL-endemic neighbouring states of Bihar and Jharkhand as also Bangladesh. A substantial proportion were inhabitants of districts in Bihar with high VL endemicity (**Fig 2**), and for professional commitments, resided in Kolkata. However, in spite of differences in their geographical origin, their demographic profiles were comparable and hence this study can be regarded as representing the overall disease scenario for PKDL, at least in India (**S1 Table**). From 2015 onwards with implementation of the elimination drive, active field surveys began in the VL/kala-azar hyper-endemic districts of West Bengal (**Fig 2)**, and proved to be a pivotal game changer, as an undetected disease burden (n = 202) was identified in only three years vis-à-vis 100 detected by passive surveillance over 15 years (**Fig 1),** akin to a study in Bangladesh where a 30 fold increase was reported [27]. The proportion of positive cases was higher through passive vs. active surveillance being 98.0 vs. 60.5%, **Fig 1,** attributable to the lower clinical proficiency of the KTS (Kala-azar Technical Supervisors) or local health workers [28], who have been trained to refer a suspected PKDL case based on a previous history of kala-azar and/or characteristic clinical features of PKDL [29]. Furthermore, majority of the negative cases (70%) detected by active surveillance presented with hypopigmented patches, which pose a diagnostic dilemma even for a dermatologist, especially as these patients reported a past history of VL **(Fig 1)**. As the diagnosis of PKDL at the field level is primarily dependent on clinical features, this is a practical problem, but it is preferable to err on the side of a positive diagnosis than leave the case neglected and allow it to be a mobile disease reservoir [4].

Following active surveillance, a sharp increase in the number of patients with macular PKDL emphasized the presence of this hidden disease burden, a sadly neglected component of a neglected tropical disease. Hypopigmented macules, especially on the face, have been reported in PKDL patients in Nepal and Bangladesh [8, 27, 30]. Till date, studies in India involving patients with PKDL, have always considered the polymorphic variant as the predominant form, ranging from 80–90% of the disease burden [7, 18, 31, 32]. Similarly, our passive surveillance recorded 77% with polymorphic lesions while only 23% presented with macular patches (**Fig 3B**). On the other hand, active surveillance reported an overwhelming increase in macular cases, the ratio of polymorphic: macular becoming almost 1:1. In view of the cumulative scenario of PKDL mimicking the active surveillance data indicating a 50:50 distribution, it is possibly a better representation of the lesional distribution of PKDL in India (**Fig 3B**).

Macular cases are less likely to seek treatment than those with disfiguring nodular and polymorphic lesions [33], **Fig 5A and 5B**. An ongoing debate regarding the role of macular cases in disease transmission was resolved by Molina et al., 2017 [26] who in a proof-of-concept experiment established that both maculopapular and nodular PKDL lesions played a definitive role in transmission, thus countering the conventional belief that macular and papular forms pose a lesser threat than nodular PKDL. Furthermore, quantification of parasite load substantiated that macular PKDL harbour a considerable disease burden [**Table 2**, 12].

Facility-based studies from South Asia have consistently reported a higher incidence of VL in males than females, the scenario being similar for PKDL [4, 7, 18, 19], possibly attributable to differences in care-seeking behaviour, males being accorded preferential treatment, females covering their lesions and therefore ignoring them, along with easier accessibility for males to reach healthcare facilities [34] whereas data from active surveillance radically differed (**Fig 3A**). Another notable feature secondary to active surveillance was the decrease in disease duration, possibly secondary to the awareness raised by the elimination drive. Contrary to previous studies where the mean disease duration was more than 5 years [8, 18, 31], active surveillance translated into an increase in the proportion of patients with a disease duration of less than a year (**Table 1**). All other features related to the disease profile remained unchanged, namely median age and the time interval between cure from VL and onset of PKDL (**Table 1**).

Although the most definitive diagnostic approach in PKDL would be parasite detection in skin smears, it has an unacceptably low detection rate ranging from 4–58%. In VL, molecular monitoring of parasites has been effective in detecting asymptomatic VL [35] and monitoring treatment efficacy [35] whereas studies in PKDL were limited, primarily due to the limited number of cases and logistic limitations for follow up. The ITS-1 PCR and LAMP (loop mediated isothermal amplification) have been successfully employed for detection of parasite DNA [12, 13, 36, 37]. However, studies pertaining to monitoring treatment effectiveness via qPCR remains limited wherein the efficacy of Miltefosine was confirmed whereas LAmB demonstrated poor efficacy [12]. For macular PKDL, their hypopigmented lesions take a considerable time to recede and therefore, monitoring the treatment efficacy of macular PKDL by clinical features is challenging. Accordingly, establishment of a ‘point of cure’ is particularly relevant [12, 38].

In the present study, the parasite burden was substantially higher in patients self reporting as compared to active surveillance (**Table 1, Fig 4A**), attributable to the reluctance of PKDL patients to actively seek treatment. This translates into them harboring the parasite for an unacceptably longer duration and being ‘mobile disease reservoirs’, and a stumbling block for the elimination efforts. Overall, irrespective of the type of surveillance, the parasite burden showed a wide IQR from 918 to 47738, comparable with previous reports [37], and the polymorphic variant consistently showed a significantly higher parasite load *vis-a-vis* the macular group (**Fig 4A, Table 2**), corroborating with other studies [36, 39]. During the disease time span of 0–12 months, no significant differences in parasite kinetics were observed between the two variants (**Fig 4B**). However, as the disease duration increased to 1–2 years and >2 years, significant differences appeared between the polymorphic and macular variant (**Fig 4B**), suggesting that differences in the host-parasite immune response may play a role in development of these two variants, emphasizing the need to perform longitudinal surveys with sequential assessment in a cohort of PKDL cases.

In a retrospective cohort study in Nepal [30], inadequate SAG treatment for VL was proposed to increase the risk of subsequently developing PKDL [21]. Irrespective of the mode of recruitment, majority of individuals in this study had received SAG as treatment for VL (**Table 3**), as unlike Bihar, West Bengal was considered as a SAG-sensitive zone. Further studies are warranted as other contributory factors e.g. inadequate SAG treatment and a lack of compliance can also increase the risk of PKDL. The appearance of PKDL has been reported with all anti-leishmanial drugs [40, 41], and even in patients with no history of VL. Using an amastigote-macrophage model, it was demonstrated that PKDL isolates were more tolerant towards miltefosine as compared to VL isolates [42], and testing of miltefosine susceptibility was recommended. In terms of the immune response generated by these drugs, SAG is more parasiticidal in nature whereas Miltefosine exerts both parasiticidal and immunomodulatory properties [43].

In 2012, WHO published a roadmap on neglected tropical diseases, including the regional elimination of VL in the Indian subcontinent by 2020 [44]. With PKDL being the disease reservoir, this study delineates the significant changes in the demographic profile of PKDL patients in West Bengal triggered by active surveillance, and endorses the benefits of active case detection in strengthening the Kala-azar elimination drive in South Asia. Most importantly, the overwhelming increase in the number of macular cases indicates that PKDL can no longer be considered as a singular entity. Furthermore, monitoring the treatment efficacy for macular PKDL is not straightforward as repigmentation of macular lesions occurs well after parasite clearance has been achieved. Therefore, in spite of the causative parasite species being *L*. *donovani*, these two variants of PKDL present with varied clinical features, suggesting possible differences in their host-parasite interactions, necessitating an in-depth analysis of the macular variant, a neglected component of this neglected tropical disease.

## Supporting information

S1 Checklist STROBE checklist. (DOCX)

S1 Table Demographic profile of patients with PKDL recruited through passive surveillance based on geographical origin. (DOCX)
