# Supplementary material for: Active surveillance identified a neglected burden of macular cases of Post Kala-azar Dermal Leishmaniasis in West Bengal
Source: PLoS Negl Trop Dis. 2019 Mar 11;13(3):e0007249. doi: 10.1371/journal.pntd.0007249 (PMC6428339; doi:10.1371/journal.pntd.0007249)
Supplement: S1 Table — (DOCX) [file pntd.0007249.s002.docx]

|  | **Passive surveillance**  **N=100** | | | |
| --- | --- | --- | --- | --- |
|  | **Overall**  **(n=100)** | **West Bengal**  **(n=35)** | **Bihar**  **(n=54)** | **Others**  **(n=11)** |
| **Age (years)*** | 25(18-35) | 26(21-48) | 23(16-38) | 24(12-45) |
| **Sex (M:F)** | 3.5:1 | 3.4:1 | 3.1:1 | 3.3:1 |
| **Disease duration (years)*** | 3(1-6) | 5(2-7) | 3.5(2-5) | 4(3-8) |
| **Lag period (interval between cure of VL and onset of PKDL, years)*** | 4(2-6) | 3(2-10) | 4.2(2-7) | 4.5(3-5) |
| **Lesion type**  **(Polymorphic: Macular)** | 77:23  (3.3:1) | 28:7  (4:1) | 41:13  (3.1:1) | 8:3  (2.7:1) |
| **Parasite load (parasites/μg of genomic DNA)*** | 17828  (2271-69159) | 27842  (6641-75542) | 31246  (3458-69475) | 36554  (6645-78849) |

**S1 Table: Demographic profile of patients with PKDL recruited through passive surveillance based on geographical origin**

*Values given in median(IQR); M: Male; F:Female
